# Flexible controls of scattering clouds using coding metasurfaces

**DOI:** 10.1038/srep37545

**Published:** 2016-11-25

**Authors:** Shuo Liu, Tie Jun Cui

**Affiliations:** 1 State Key Laboratory of Millimeter Waves, Southeast University, Nanjing 210096, China; 2 Synergetic Innovation Center of Wireless Communication Technology, Southeast University, Nanjing 210096, China; 3 Cooperative Innovation Centre of Terahertz Science, No. 4, Section 2, North Jianshe Road, Chengdu 610054, China

## Abstract

Metamaterials or metasurfaces have been designed to precisely manipulate the scattering at every angle. Here, we propose to control the probability of random scattering appearing in the desired range of angles, which is defined in this letter as scattering cloud. We present a controllable random metasurface by simply adding a random coding sequence to gradient coding sequence. It is shown that the direction and size of the scattering cloud can be arbitrarily engineered. We demonstrate the exotic behavior of the scattering cloud by making an analogy to the electron cloud in quantum mechanics. A new coding particle featuring low-interference with neighboring coding particles is designed to realize the controllable random surface, which demonstrates highly consistent results to the theoretical calculations using fast Fourier transform. The exciting phenomena and versatile behaviors of scattering clouds and their probabilities enabled by controllable random surfaces will lead to diversified applications in the fields of electromagnetic waves and acoustic waves.

In the past decade, the manipulation of electromagnetic (EM) wave has gradually evolved from three-dimensional (3D) metamaterial^1^,^2^,^3^ to two-dimensional (2D) metasurface^4^, which is composed of periodically or non-periodically arranged subwavelength scatterers on a deep subwavelength surface. Compared with 3D bulk metamaterials, the significant reduction in the thickness of metasurfaces allow them to control EM waves with less metal and dielectric absorptions and lighter weight. Due to their planar configuration, metasurfaces can be easily implemented at the terahertz and visible light spectra using the standard photolithography process, and have led to development of many practical devices including perfect absorber^5^,^6^,^7^, polarization convertor^8^,^9^,^10^, modulator^11^,^12^,^13^, and holography^14^,^15^,^16^. The manipulation of the wavefront for bending and focusing light using a single^18^,^19^ or multiple layers^10^,^20^ of metallic strcutures with elaborately designed phase distributions has received increasing attention since the proposal of the generalized Snell’s law^17^ in 2011 to reflect/refract the normal incidence in anomolous directions.

In 2014, the concept of coding metasurface^21^ was proposed by designing two subwavelength coding particles (unit cells) ‘0’ and ‘1’ with opposite reflection phases, respectively. By simply arranging these coding particles with pre-designed coding sequences on a flat surface, a variety of functionalities of the coding metasurface have been demonstrated in microwave and terahertz frequencies, such as the anomalous beam reflections^21^,^22^, random diffusions^23^,^24^, and polarization conversion^22^. Similar to the digital circuit, where the quantization of the analogue signal into binary states “on” and “off” enhances the signal-to-noise ratio (SNR) effectively, the discretization of the continuous phase profile can help increase the robustness of the coding metasurface against environment changes in practical applications, such as the deviation of the reflection responses due to fabrication errors of the structure and the chemical contamination with different refraction indices. Most importantly, coding metasurfaces with binary codes have provided great convenience to explore relations between the coding pattern and radiation pattern from the fully-digital perspective, resulting in digital and programmable metasurfaces^21^. Many existing theorems from the information science, such as fast Fourier transform (FFT) and information entropy^25^,^26^, can be directly applied in the analyses of coding metasurfaces. Here we should note that our coding metamaterial is different from the digital metamaterial proposed by Engetha^27^, which is introduced to design new materials whose permitivity can be artitrarily controlled by mixing two different materials (named as metamaterials bit) in certain proportions (named as the metamaterials byte).

## Results

### Concept of controllable random surface

**Figure 1:**
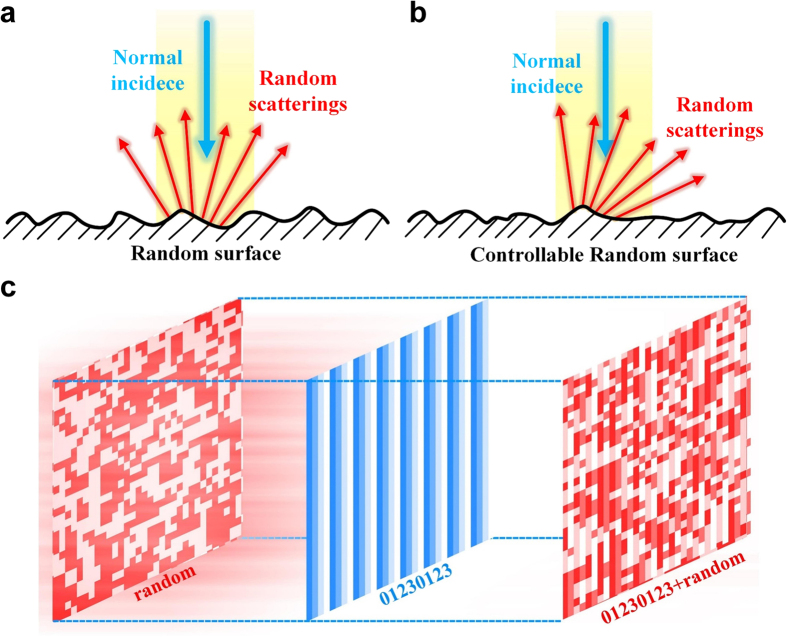
Conceptual illustrations of the conventional random surface and the proposed controllable random surface. (**a**,**b**) Schematics for the conventional random surface and the proposed controllable random surface. (**c**) Schematic illustration for the generation of the mixed coding pattern (the right pattern) of the controllable random surface from the addition of a periodic coding sequence “01230123…” (the middle pattern) and a random coding sequence (the left pattern).

In previous coding schemes for broadband diffusions of EM waves using randomly distributed Minkowski-loop^23^ and square-patch^21^ coding particles, the incident beam is simply redistributed in numerous directions in the entire upper-half space. This type of conventional random surface^28^,^29^, known as rough surface at visible light spectra, is schematically illustrated in Fig. 1a, where the normally incident light rays (blue arrows) are randomly scattered by the random surface in multiple directions (red arrows). Although the radar cross section (RCS) of a flat or cylindrical metallic object, especially the strong reflection in the back scattering direction, can be effectively reduced by such random surfaces, we are not able to predict, or to control, the directions of scattered waves. Here, we propose a new concept of controllable random surface, which is unlike any other rough surfaces from natural materials. The random scattering diffused by the controllable random surface can be manipulated in a desired manner in terms of the locations in the upper-half space, such as the main directions and the angle ranges of each main direction. This concept is illustrated in Fig. 1b, in which the random scattering that is originally located around the surface normal is diverted to the right-hand side by the elaborately designed controllable random surface. We remark that, unlike the previous demonstrated periodic coding sequences to generate a single or multiple beams pointing in definite directions, the controllable random surface is not designed to precisely manipulate the scattering at every angle, but to control the probability of random scattering appearing in desired range of angles, which is defined in this paper as scattering cloud. In this regard, the scattering cloud can be compared to, in a sense, the electron cloud in an atom, where one can never know the accurate position of the electron but only its probability inside a certain spherical zone, according to Heisenberg uncertainty principle in quantum mechanics.

As is known from the previous work, a gradient periodic coding sequence “01230123…” from a 2-bit coding metasurface could generate a single-beam scattering pointing in a certain direction^21^, whereas the random scattering from random coding sequence is roughly located within a certain range of angles around the surface normal^21^,^22^,^23^,^24^. Then, an interesting idea arises: what happens if we add a periodic coding sequence to a random one? This conception is illustrated in Fig. 1c, where a random coding pattern (on the left) and a periodic coding pattern with “01230123…” gradient coding sequence (in the middle) are added together, resulting in their modulus (mod by 4) on the right side. It is interesting to find that the mixed coding pattern, which forms the controllable random surface, possesses both features of the random and the periodic coding patterns. For this reason, one may intuitively expect both characteristics: the random diffusion and single beam scattering, from the mixed scattering pattern.

### Theoretical characterization

**Figure 2:**
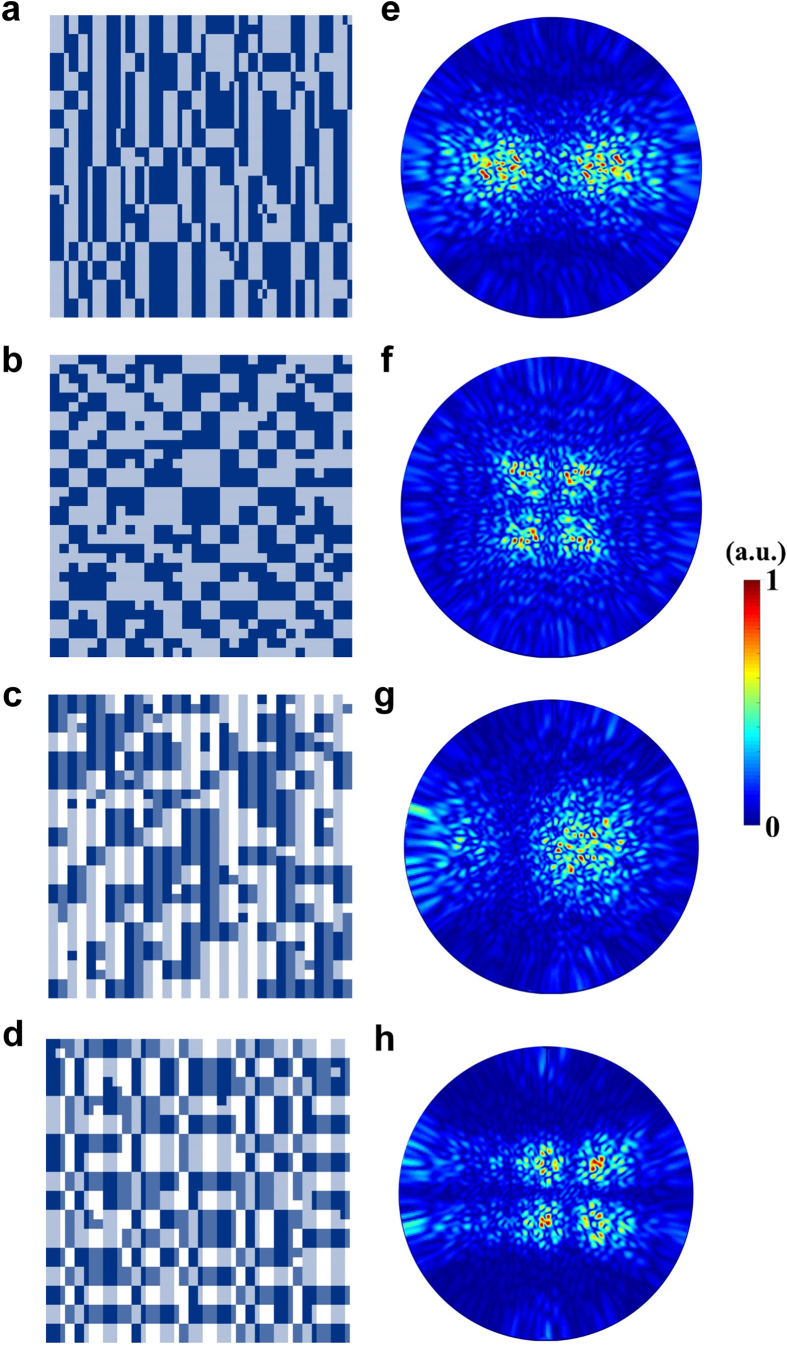
Performance of the controllable random surface in diffusing the EM wave to desired directions. (**a**–**d**) The mixed coding patterns M_1_–M_4_. (**e**–**h**) The 3D scattering patterns for M_1_–M_4_ plotted in the polar coordinate, where the radial and azimuthal directions represent respectively, the elevation angle *θ* and azimuthal angle *φ* in the spherical coordinate.

To demonstrate the unusual physical phenomena of the controllable random surface, we first consider the 1-bit coding metasurface composed of only two coding digits “0” and “1”. Figure 2a shows the coding pattern M_1_, which includes 64 × 64 coding particles and is obtained by mixing a random coding sequence and a periodic “0101…” sequence. The depth of the blue color indicates the value of coding digit. To mix the random coding pattern and the periodic coding pattern, we first add them together and then calculate the modulus of it by 2. Each coding digit in the periodic coding sequence is composed of *N* × *N* identical coding particles, defined in the previous works as the super-unit-cell^21^. Table 1 lists the detailed information of the periodic coding sequences for the coding patterns M_1_–M_4_. Here, we provide a fast and efficient method for calculating the 3D scattering pattern directly from the coding pattern. Please refer to the method section for the details of the generation of mixed coding pattern and the procedure of fast calculation of the scattering pattern.

Figure 2e shows the 3D far-field scattering pattern plotted in the polar coordinate, in which the radial and azimuthal directions represent the elevation angle *θ* and azimuthal angle *φ* in the spherical coordinate, respectively. All the scattering patterns have been normalized to their maximum intensity. It can be observed that most of the random scatterings appear in the left and right circular areas, with their center exactly located at the radiation directions of the periodic “0101…” sequence. The accurate value of the those directions can be theoretically calculated by the function of the generalized Snell’s law 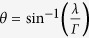
22 as (*θ* = 30°, *φ* = 0°/180°), in which *λ* and *Γ* are the working wavelength and periodicity of the periodic coding sequence. Because the above- described directions are determined by the periodicity of the periodic coding sequence, we could divert the random scattering to larger angles by decreasing the periodicity, or vice versa. Figure 2b shows the coding pattern M_2_, which is obtained by mixing a random coding sequence with a periodic coding sequence that features the chessboard distribution. As expected, the random scattering shown in Fig. 2f is split into four regions, with their centers pointing in the radiation directions of the chessboard distribution coding sequence (*θ* = 32°, *φ* = ±45°/135°).

In certain applications, the random scattering may be required to be diverted to one direction. This can be realized by the 2-bit coding metasurface which allows much more pattern flexibility. Figure 2c shows the coding pattern M_3_ that is mixed by a random coding sequence and a periodic coding sequence “01230123…”. The corresponding 3D scattering pattern is shown in Fig. 2g, where an obvious scattering cloud can be found to the right side of the center, around (*θ* = 30°, *φ* = 0°).

One may be interested in what the scattering pattern would be like if a random coding sequence is added with two different periodic coding sequences, such as “01230123…” and chessboard distribution. Note that, when the 1-bit and 2-bit coding digits are added together, the 1-bit coding digits should be multiplied by two in order to keep the 180° phase difference (i.e. “0101…” should be converted to “0202…”). And then we should calculate the modulus of them by 4. The resulting coding pattern M_4_ from such a combination is shown in Fig. 2d. Since it can be viewed as the addition of the coding pattern M_3_ with a periodic coding sequence “01230123…”, we would expect a similar scattering pattern as Fig. 2f but is shifted to the right side. This can be verified from the 3D scattering pattern shown in Fig. 2h.

To further elaborate the physical mechanism of such a rotation of the random radiation pattern to the desired directions, we would like to make an analogy to the mixing process (modulation) in signal processing. In fact, there is a Fourier transform relation between the coding pattern (near-field distribution) and the far-field radiation pattern. Therefore, any property from the Fourier transform can be directly applied to the design of coding metasurface. As we know, a carrier wave with single frequency can be viewed as an ideal Dirac function from the frequency-domain perspective. The frequency spectrum of a baseband signal can be shifted to the central frequency of the carrier wave without distortion, after being multiplied with the carrier wave in the time-domain, or conducted by a convolution operation on its spectrum (Dirac function) in the frequency domain. For coding metasurfaces, the gradient coding sequence “01230123”, with unity amplitude and linearly increasing phase profile, can be viewed as a carrier wave with single frequency in the time domain, or Dirac function in the frequency domain. Hence the random coding pattern, which is compared to the baseband signal, can be steered to an oblique direction with little distortion in the same manner as the above mixing (modulation) process.

**Figure 3:**
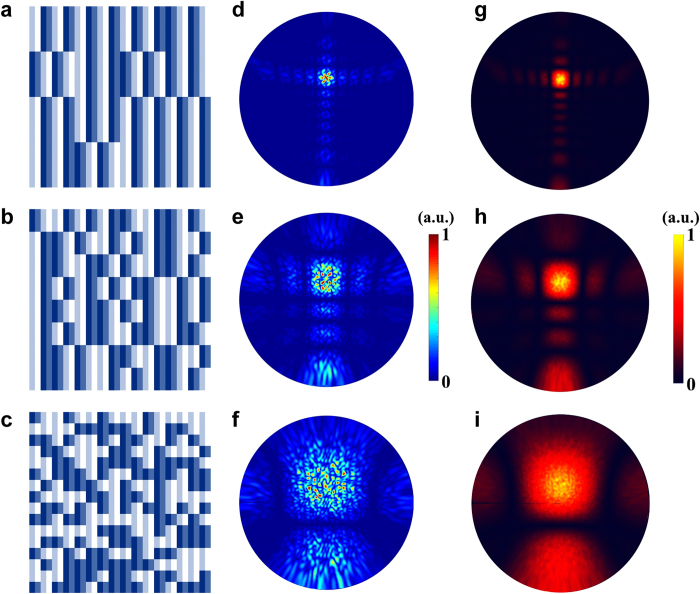
Manipulation of the level of scattering diffusion by controlling the super-unit-cell size of the controllable random surface. (**a**–**c**) The mixed coding patterns when the weight numbers ***w*** of the three random coding patterns are set as ***w***_***1***_ = [0 1 0 0 0 0], ***w***_***2***_ = [0 0 1 0 0 0] and ***w***_***3***_ = [0 0 0 1 0 0]. (**d**–**f**) The corresponding 3D scattering patterns. (**g**–**i**) The corresponding plots for the probability clouds of the random scatterings.

The above cases have demonstrated the powerful control on the directions of the scattering cloud by simply adding one or more periodic coding sequences with a random one. Here, we show that the area of scattering could also be manipulated by altering the size of super-unit-cell in the random coding pattern. To demonstrate the influence of super-unit-cell size on the area of scattering cloud, we add a periodic coding sequence “01230123…” (super-unit-cell size is 2 × 2) with three random coding patterns, each having a distinct super-unit-cell size. Figure 3a–c show the mixed coding patterns when the weight arrays ***w*** (see the method section) of the three random coding patterns are set as ***w***_***1***_ = [0 1 0 0 0 0], ***w***_***2***_ = [0 0 1 0 0 0], and ***w***_***3***_ = [0 0 0 1 0 0], respectively.

For the first case, since the super-unit-cell size of the random coding matrix 16 × 16 is the largest among the three cases (see Fig. 3a), the random scattering is confined in a small area, as is observed from the 3D scattering pattern in Fig. 3d. As the size of super-unit-cell further reduces to 8 × 8, the mixed coding pattern becomes more randomly distributed, as shown in Fig. 3b. Consequently, the EM energy is diffused to a larger range of directions (see Fig. 3e), compared to the first case (see Fig. 3d). For the last example where each super-unit-cell is composed of 4 × 4 identical coding particles, we have the largest number of random coding digits (16 × 16 = 256) in the random coding pattern (see Fig. 3c). As expected, the scattering cloud covers the biggest area among the three cases, as illustrated in Fig. 3f.

This example reveals the relation between the level of diffusion effect (i.e., the area of a single scattering cloud) and the super-unit-cell size of the random coding pattern (i.e., the number of random coding digits). This finding enables us to control the size of scattering cloud flexibly by changing the size of super-unit-cell in random coding pattern. Smaller super-unit-cell size generates scattering cloud with larger area, which implies higher uncertainty of scatterings appearing in a fixed range of angles in the upper-half space. Once the super-unit-cell size is given, we could obtain a series of coding patterns that generate the same-sized scattering clouds. Although these scattering patterns may look different from each other, the probability of scattering appearing at a certain direction with certain intensity is definite. This feature is quite similar to the experimental observation of electrons in an atom. Even though the electron may appear at different positions in each observation, one should get the same probability distribution of the electron’s position in the atom if the experiment is repeated for enough times. This principle can also be found in the controllable random surface. Figure 3g–i show the corresponding probability clouds of random scattering for weight arrays ***w***_***1***_–***w***_***3***_, each obtained from the averaged result of 100 different scattering patterns that have the same super-unit-cell size. Clearly, the bright level in each plot indicates the relative probability of random scattering appearing at a specific direction. That is to say, the random scattering should have more chances to appear at those directions with brighter color. The center points of probability clouds for all three cases coincide because they share the same periodic coding sequence. Again, the size of the probability cloud gradually increases as expected with the decreasing of the super-unit-cell size of the random coding sequence. Please find more details about the relationship between the level of scattering diffusion and super-unit-cell size in Supplementary Information Figure S1.

Here, we find an interesting connection between the potential information carried by a controllable random surface and size of scattering cloud by analyzing the controllable random surface from the perspective of Shannon entropy^25^,^26^, which is used to measure the unpredictability of information content. We know that a signal source with larger entropy (i.e. higher uncertainty) should theoretically be able to deliver more information than that with smaller entropy (i.e. less uncertainty). Likewise, a radiation pattern with random scattering carries more information than that with a single or only a few scattering beams. That is to say, more random coding digits (i.e. smaller super-unit-cell size) result in more uncertainty of the scattering that appears in a certain direction, and thus possessing potentially more information. This could be better understood by the application of a single-sensor imaging system^30^. In order to retrieve the image of an object with high resolution and accuracy, i.e. with more information, the scattering pattern generated from the transmitting antenna is required as random as possible. With the controllable random surface, we could easily obtain a series of coding patterns that have the same information entropy, which might be of particular interests in the applications like compressive radar imaging^31^.

### Numerical simulation

**Figure 4:**
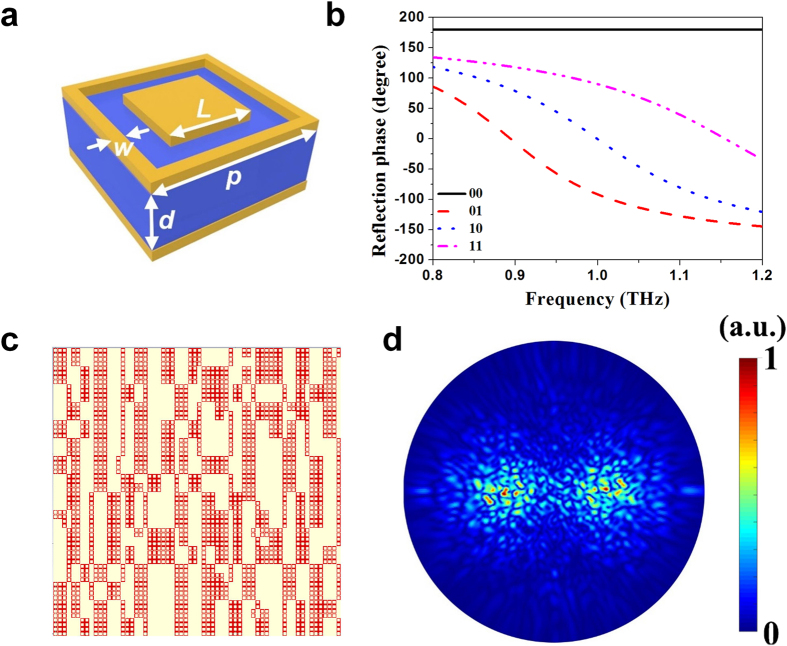
Numerical implementation for the controllable random surface using a low-interference structure. (**a**) The structure of the coding particle design. (**b**) The simulated phases of reflections for the four coding particles from 0.8 to 1.2 THz. (**c**) The model of the coding pattern M_1_ built in CST. (**d**) The numerically simulated scattering pattern for M_1_.

In order to implement the controllable random surface with a realistic structure, we design the coding particle as shown in Fig. 4a, which features low EM interference with adjacent coding particles due to the existence of the metallic frame added to the edge of each coding particle. The design details for this structure and the comparison of it with structures in the previous works^21^,^22^,^23^,^24^ are described in the Supplementary Information. Figure 4b shows the simulated phases of reflections from 0.8 to 1.2 THz of the four optimized coding particles, which reach exactly to −180°, −90°, 0°, and 90° at the operating frequency 1.0 THz. The amplitudes of all four coding particles remain above 0.75 at the designed frequency (see Supplementary Information Figure S2).

We built the coding pattern M_1_–M_4_ in CST using the designed structure (see Fig. 4c for the coding pattern M_1_) and simulated them with the time-domain solver. It is clearly observed from Fig. 4d that the numerically simulated scattering pattern for M_1_ is highly consistent with the theoretically calculated one given in Fig. 2a. Excellent agreements can also be found between the numerically simulated scattering patterns for M_2_–M_4_ (see Supplementary Information Figure S3) and the theoretical results in Fig. 2f–h, which are due to the low-interference characteristic of the designed structure. Such a highly consistency between the numerical simulation and theoretical calculation validates the feasibility of the approach to use coding metasurface for the implementation of the controllable random surface, and at the same time, verify the effectiveness of the method for the fast calculation of the scattering patterns using FFT.

In addition to the outstanding performance, the controllable random surfaces are especially appealing for their flat profile and ease of fabrication with respect to 3D bulk structures. We remark that they may have potential applications not only in the terahertz frequency, but also in the microwave or visible light spectra, or even for the acoustic waves. Some of the possible applications that may benefit from this concept are discussed as follows.

For the single-sensor imaging system in the microwave or millimeter wave frequencies, the random scattering generated by the transmitting antenna is usually confined in a certain range of angles with respect to the optical axis, fundamentally limiting the area that could be effectively imaged at the imaging plane. With the controllable random surface, however, the random scattering can be deviated to larger oblique angles, and therefore could increase the size of image area. At the visible light spectrum, a material with artificially tailored diffusion may have a wide range of applications in the fields where the conventional reflective/diffusion materials are applied, such as warning board, facade material of building, clothing materials, etc. For instance, it can be used to replace the reflective material around the bulb to guide the light in the desired range of directions, or to confine the diffusion of the projection screen in the audience direction to improve the luminance. The concept of controllable random surface can also be extended to acoustic waves^32^,^33^, and may promise many novel acoustic diffusion materials with unusual diffusion properties.

## Conclusion

In this work, inspired by the functionalities of random scattering and beam scanning capability of coding metasurfaces, we proposed the concepts of controllable random surface and scattering clouds by combining periodic and random coding sequences. It is interesting to remark that the scattering cloud behaves like the electron cloud in an atom in the aspect that every definite coding pattern of the controllable random surface is essentially a particular instance of the probabilistic model (probability cloud) under a certain control parameter (e.g. the size of super-unit-cell). Moreover, just like the superposition principle of quantum mechanics that wave functions can be added together and multiplied by complex numbers to form new wave functions, the shape of the scatterings cloud, which is determined by the probabilistic model of the controllable random surface, can be tailored in a similar manner by superposing multiple random coding sequences with different weights, and various periodic coding sequences.

As an example of practical implementation of the controllable random surface using realistic structures, we designed and simulated a novel coding particle that features low EM interference with neighboring unit cells. The numerical simulations agree very well with theoretical calculations. Given the exotic physical phenomena of the controllable scattering clouds and their probabilities, our design can be considered, in a sense, a new type of diffusion material that may have a great impact on the conventional reflective and diffusion materials from microwave to optical spectra, and even for acoustic waves.

## Method

### Generation of the random coding pattern

The random coding pattern M_*random*_ is formed by the following function:


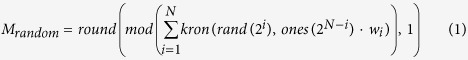


in which the item *kron(rand*(2^*i*^), *ones*(2^*N*−*i*^) represents a 2^*i*^ × 2^*i*^ partitioned matrix, and each partitioned matrix is composed of 2^*N*−*i*^ × 2^*N*−*i*^ identical random numbers on the open interval (0,1); *w*_*i*_ is the weight (random number on the open interval (0,1)) on each of the above random matrix. According to Equation (1), the final random coding pattern of the controllable random surface is obtained by the following setup. First, sum up these different random matrices as the integer i ranges from 1 to N; then calculate the modulus of it by one; finally round the continuous random numbers (from 0 to 1) to the discrete binary codes “0” and “1”. In this work, N is chosen as 6, making a coding pattern with 64 × 64 coding particles. The weight array **w** = [*w*_1_, *w*_2_, *w*_3_, *w*_4_, *w*_5_, *w*_6_] is determined randomly for the following four cases.

### Procedure of calculating the 3D scattering pattern

The procedure for the fast calculation of the 3D scattering pattern directly from the coding pattern mainly includes two steps, which are a 2D FFT of the coding pattern and a coordinate transformation from the angular coordinate (*u, v*) to the visible angle coordinate (*θ, φ*) using the following equations,


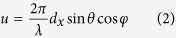



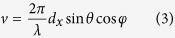


in which *d*_*x*_, *d*_*y*_ are the periodicities of the coding particle that are distributed on the 2D plane along the *x* and *y* directions, respectively. λ = 300 μm is the working wavelength. As (*u, v*) range from 0 to π, *d*_*x*_, *d*_*y*_ should be less than half wavelength so that (*θ, φ*) can cover all the visible angles, which means the encoded metasurface will have the capability to scan in the entire upper-half space. During the theoretical calculation, we have assumed the amplitude as unity across the square area (100 × 100 μm) of each coding particle, and their phases as the ideal 0° and 180°. We remark that the computational time is dramatically reduced to only several seconds, at least three orders of magnitude fewer than that took by the numerical simulations using the commercial software, CST Microwave Studio.

## Additional Information

**How to cite this article**: Liu, S. and Cui, T. J. Flexible controls of scattering clouds using coding metasurfaces. *Sci. Rep.* **6**, 37545; doi: 10.1038/srep37545 (2016).

**Publisher’s note:** Springer Nature remains neutral with regard to jurisdictional claims in published maps and institutional affiliations.

## Supplementary Material

Supplementary Information

## Figures and Tables

**Table 1:** Information of the periodic coding sequences for the coding patterns M_1_–M_4_.

Coding pattern (periodic part)	Combination	Size
M_1_	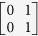 (N = 3) 1-bit	64 × 64
M_2_	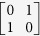 (N = 3) 1-bit	64 × 64
M_3_	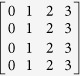 (N = 2) 2-bit	64 × 64
M_4_	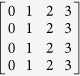 (N = 2) 2-bit+ 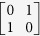 (N = 3) 1-bit	64 × 64
