# Supplementary material for: Flexible controls of scattering clouds using coding metasurfaces
Source: Sci Rep. 2016 Nov 25;6:37545. doi: 10.1038/srep37545 (PMC5122876; doi:10.1038/srep37545)
Supplement: Supplementary Information [file srep37545-s1.pdf]

Supplementary Information for

Flexible controls of scattering clouds using coding metasurfaces

Shuo Liu[1,2] and Tie Jun Cui[1,3+]

[1] State Key Laboratory of Millimeter Waves, Southeast University, Nanjing 210096, China
[2] Synergetic Innovation Center of Wireless Communication Technology, Southeast University, Nanjing 210096, China
[3] Cooperative Innovation Centre of Terahertz Science, No.4, Section 2, North Jianshe Road, Chengdu 610054, China

[+] Corresponding author:

E-mail: tjcui@seu.edu.cn.

Tel: 008683790295

Fax: 008683790295

This PDF file includes:

The detailed descriptions of 1) The relationship between diffusion effect and super-unit-cell size; 2) Numerical verification and 3) Figures S1-S3.

1. The relationship between diffusion effect and super-unit-cell size

To characterize the relationship between the level of scattering diffusion and super-unit-cell size, we further calculate the probability clouds for the coding pattern that has only the random part, which are shown in Figures S1a-c for weight arrays $\boldsymbol{w_1}$-$\boldsymbol{w_3}$, respectively. Same as the probability clouds in Figure 3, each probability cloud in **Figure S1**a is obtained from the averaged result of 100 different scattering patterns that have the same super-unit-cell size. It is clear that the random scatterings gradually diffuse to larger solid angles as the super-unit-cell size decreases. Figures S1d-f show the 2D probability clouds on the vertical cutting plane for weight arrays $\boldsymbol{w_1}$-$\boldsymbol{w_3}$, respectively. We notice that the in all three cases, the scattered electric fields decrease almost linearly from 0° to the angle where the curve reaches the first null. This means that the probability of the appearing of random scatterings decreases almost linearly with the increasing of elevation angle. The blue area in each plot outlines the angle range calculated by function $\theta = \sin^{-1}\left(\frac{\lambda}{\Gamma}\right)$, where Γ equals 3200 μm, 1600 μm and 800 μm for weight arrays $\boldsymbol{w_1}$-$\boldsymbol{w_3}$, respectively. It is found that the intensity of electric field inside this angle range is over 55-60% of the peak value (around the 0° direction). Hence, for a random coding pattern with certain super-unit-cell size, the above function can be used to estimate roughly the angle range where the intensity of scattered electric field is larger than 55-60% of the peak value.

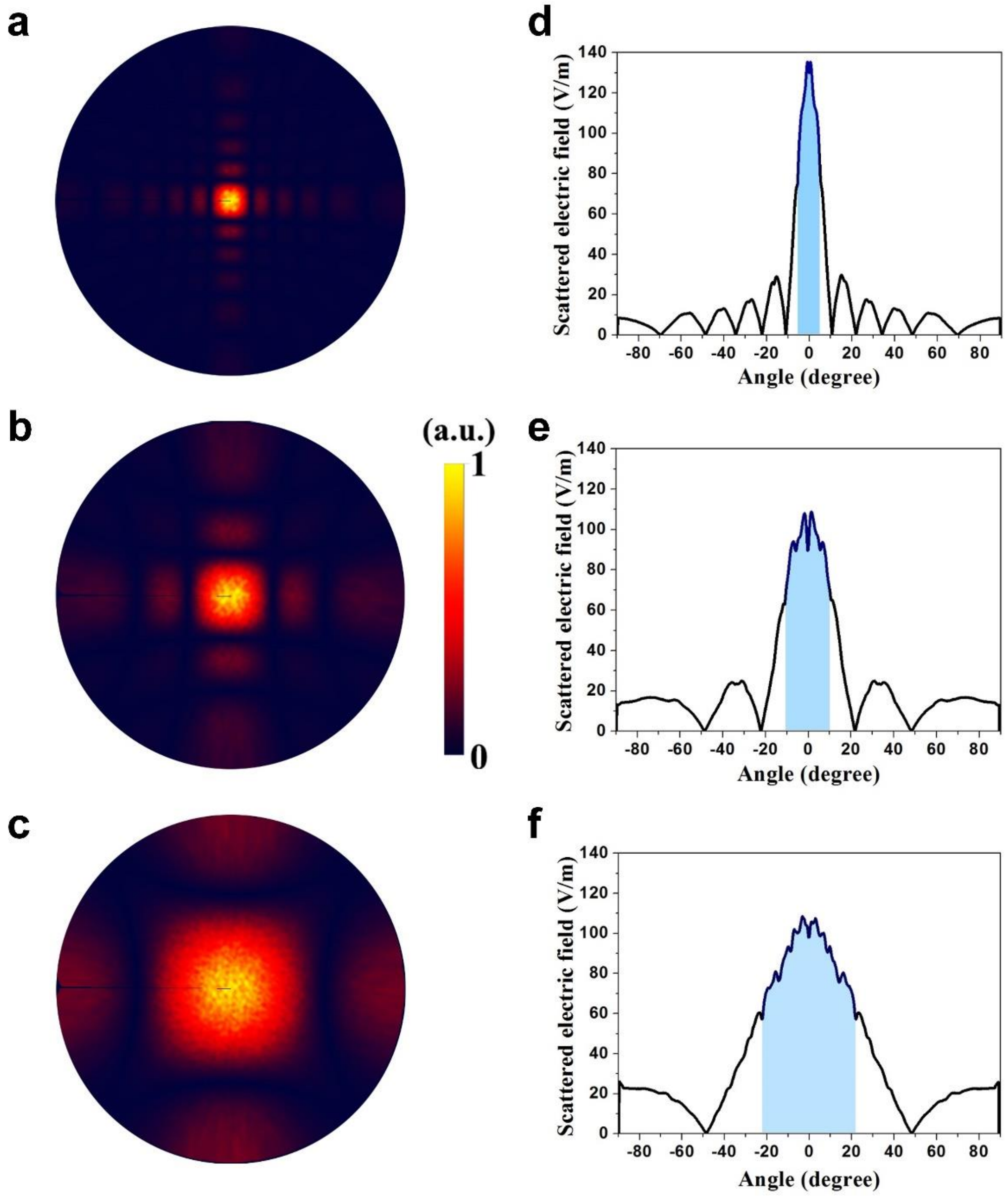

Figure. S1 The relation between the diffusion effect and super-unit-cell size. (a-c) The plots for the probability clouds when the weight numbers $\mathbf{w}$ of the three random coding patterns are set as $\boldsymbol{w_1}$=[0 1 0 0 0 0], $\boldsymbol{w_2}$=[0 0 1 0 0 0] and $\boldsymbol{w_3}$=[0 0 0 1 0 0]. (d-f) The corresponding 2D probability clouds on the vertical cutting plane. The blue area in each plot outlines the angle range calculated by function $\theta = \sin^{-1}\left(\frac{\lambda}{\Gamma}\right)$, in which the intensity of scattered electric field is larger than 55-60% of the peak value

2. Numerical verification

The designed coding particle in Figure 4(a) is composed of a metallic pattern and a metallic background, separated by a polyimide spacer (ε_r=3, δ=0.03) with thickness d=25 μm. For the square patch structure proposed in our previous work [1], the coding particles at the edge of each super-unit-cell may not provide ideal reflection phases because the reflection response highly relies on the distance between adjacent patches. This is one of the primary concerns of the coding metasurface that substantially affect the performance of the radiation pattern. A possible solution to suppress such undesired EM couplings is to increase the size of super-unit-cell, as has been adopted in the previous works [1-4]. With this approach, however, the radiation beam will be fundamentally limited to a small range of angels (elevation angle θ) with respect to the normal axis when the size of super-unit-cell is set too large.

Using the frequency-domain solver of the commercial software, CST Microwave Studio, we obtain the optimized parameters L as 100, 71.9, 64.7 and 55.2 μm for the four coding particles "00", "01", "10", and "11", respectively. **Figure S2** shows the amplitudes of reflections for the four coding particles. Although the reflection amplitudes of the four coding particles are different, they are larger than 0.75 and have little influence on the radiation pattern, as is verified by the following numerical simulations.

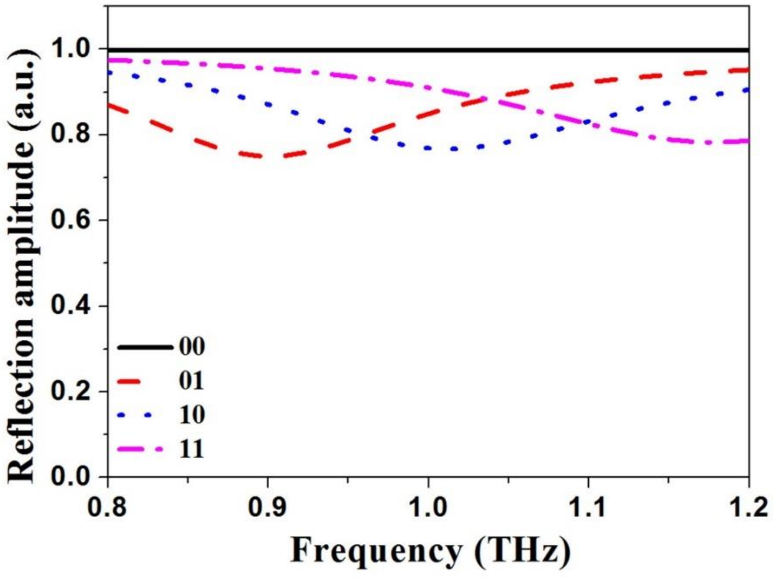

Figure. S2 The amplitudes of reflections for the four coding particles.

The numerically simulated scattering patterns in Figure 4(d) and **Figure S3** are simulated by the time-domain solver in CST with the open boundary condition. Even though the coding patterns of such four models have small super-unit-cell sizes (e.g. 2×2 and 1×1), which means the undesired EM couplings between adjacent coding particles may be significant, it is clearly observed that the numerically simulated scattering patterns in Figure S3 are highly consistent with the theoretically calculated results given in Figures 2(f)-(h).

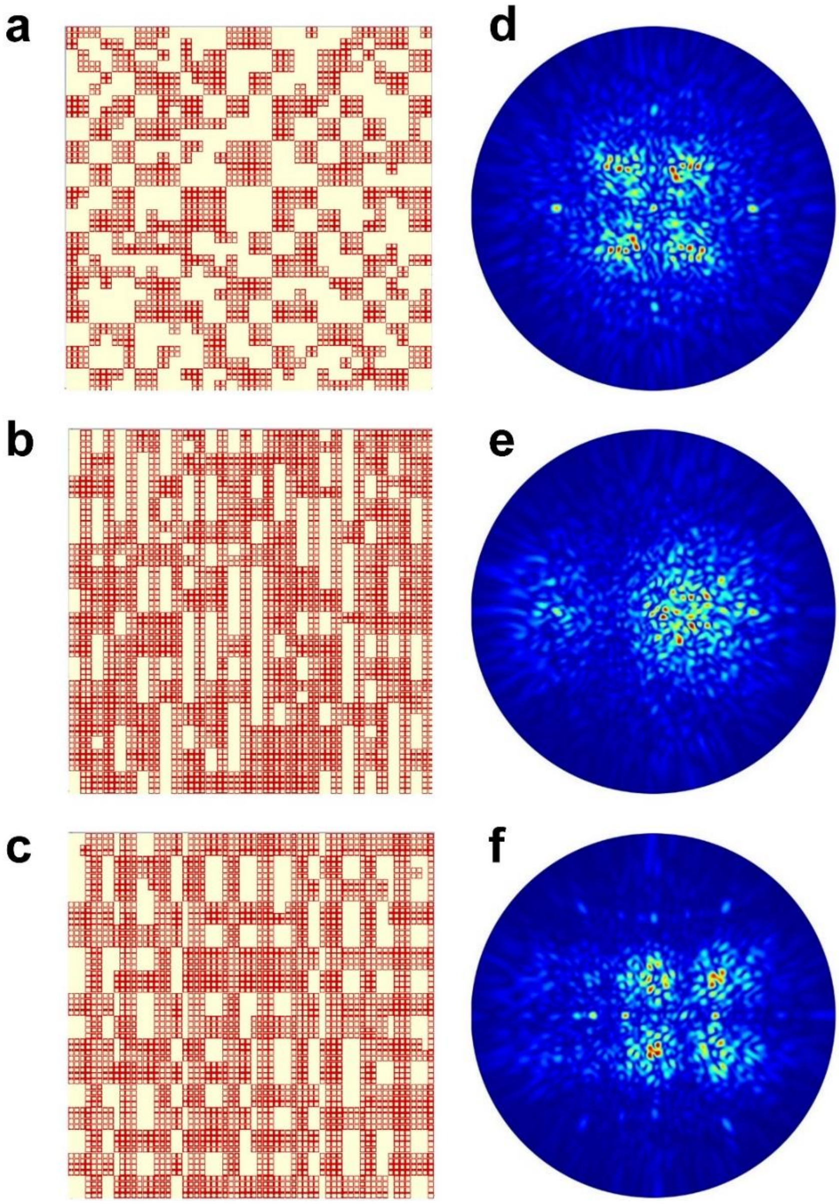

Figure. S3 Numerical verifications of the coding patterns M_2-M_4. (a-c) The models of mixed coding patterns M_2-M_4 built in CST. (d-f) The corresponding 3D scattering patterns for M_2-M_4.

References

[1] T. J. Cui, M. Q. Qi, X. Wan, J. Zhao & Q. Cheng. Coding metamaterials, digital metamaterials and programmable metamaterials. *Light-Sci. Applicat.* **10**, e218-1-e218-9 (2014).

[2] S. Liu et al. Anisotropic coding metamaterials and their powerful manipulation to differently polarized terahertz waves. *Light-Sci. Applicat.* **5**, e16076-1-e16076-11 (2016).

[3] L. H. Gao et al. Broadband diffusion of terahertz waves by multi-bit coding metasurfaces. *Light-Sci. Applicat.* **4**, e324-1-e324-9 (2015).

[4] L. J. Liang et al. Anomalous terahertz reflection and scattering by flexible and conformal coding metamaterials. *Adv. Opt. Mater.* **3**, 1374-1380 (2015).
